# Evaluation of passive immunity transfer in Danish dairy calves measured by enzyme-linked immunosorbent assay and Brix refractometer

**DOI:** 10.1186/s13028-025-00801-0

**Published:** 2025-03-29

**Authors:** Anne Marie Michelsen, Nina Dam Otten, Mogens Vestergaard, Liza Rosenbaum Nielsen, Bodil Højlund Nielsen, Henrik Laessøe Martin, Nynne Capion, Mette Bisgaard Petersen

**Affiliations:** 1 Department of Veterinary and Animal Sciences, Faculty of Health and Medical Sciences, University of Copenhagen, Grønnegårdsvej 8, Frederiksberg C, DK-1870 Denmark; 2 Department of Animal and Veterinary Sciences, Faculty of Technical Sciences, Aarhus University, Blichers Allé 20, Tjele, DK-8830 Denmark; 3 SimHerd A/S, Agro Business Park, Niels Pedersens Alle 2, Tjele, DK-8830 Denmark; 4 Seges Innovation, Agro Food Park 15, Aarhus N, DK-8200 Denmark; 5 Department of Veterinary Clinical Sciences, Faculty of Health and Medical Sciences, University of Copenhagen, Højbakkegårds Alle 5A, Taastrup, DK-2630 Denmark

**Keywords:** Bovine, Failure of transfer of passive immunity, Percentage Brix, Serum Immunoglobulin G level

## Abstract

Calves are born agammaglobulinemic and depend on transfer of passive immunity from colostrum. Failure of transfer of passive immunity (FTPI) and adequate transfer of passive immunity (ATPI) are defined as serum immunoglobulin G (IgG) levels < 10 and ≥ 10 mg/mL, respectively. The objective of this study was to evaluate the level of passively transferred immunity in Danish dairy calves measured with Enzyme-linked immunosorbent assay (ELISA) and Brix refractometer. Blood samples from 834 calves (430 bull calves and 404 heifer calves) aged two to nine days were included in the study. Serum IgG concentration was determined by ELISA and percentage Brix (%Brix) with a digital refractometer. Median serum IgG concentration was 17.05 mg/mL and mean %Brix was 8.5%. A total of 592 (71.0%) and 242 samples (29.0%) had a serum IgG concentration ≥ 10 mg/mL and < 10 mg/mL, respectively. For %Brix 541 (64.9%) and 293 (35.1%) was ≥ 8.1 and < 8.1%, respectively. Serum IgG concentrations and %Brix measurements were highly correlated (*r* = 0.88). The level of passive immunity in Danish dairy calves seems low and does not meet new recommendations. However, applying cut-offs based on radial immunodiffusion to serum IgG concentrations derived from ELISA are not straightforward and determination of new cut-off values for FTPI based on ELISA are recommended.

## Findings

Calves are born agammaglobulinemic and depend on the transfer of immunoglobulins (Ig) through ingestion of colostrum to obtain immunity [1]. The passive immunity of calves can be evaluated with different methods from 1 to 9 days of age [2].

The most widely used definition of failure of transfer of passive immunity (FTPI) is serum IgG level < 10 mg/mL and adequate transfer of passive immunity (ATPI) as ≥ 10 mg/mL [3–5]. However, a recent study suggested new recommendations on serum IgG levels including four categories: poor (< 10 mg/mL IgG), fair (10-17.9 mg/mL IgG), good (18-24.9 mg/mL IgG) and excellent (≥ 25 mg/mL IgG) [5]. These recommendations are based on IgG determination using radial immunodiffusion (RID) [6], which is considered the gold standard method for determination of IgG in calf serum [7–9]. The method is, however, time consuming and expensive [10, 11] and Enzyme-linked immunosorbent assay (ELISA) may be used as an alternative and studies have shown a high correlation between the two methods despite the fact that different absolute IgG values were found [10, 12, 13].

Estimating serum IgG levels using a digital Brix refractometer has proven to be a quick and reliable method for on-farm use [11, 14]. Like for serum IgG levels, four categories were suggested for percentage Brix (%Brix); poor (< 8.1), fair (8.1–8.8), good (8.9–9.3) and excellent (≥ 9.4) with < 8.1% Brix being equivalent to < 10 mg/mL IgG [5].

The objective of this study was to evaluate the level of passive immunity in Danish dairy calves and to compare ELISA and digital Brix refractometer for the determination of serum IgG levels.

From September 2018 to November 2019, 1002 calves in 83 Danish dairy herds were visited and sampled for different purposes as part of a large study on calf health in Denmark. The selected herds included a range of herd sizes (89–989 cows, mean herd size 318 cows) and breeds including Danish Holstein (72%), Danish Jersey (1%), Danish Red (5%) and dairy crossbreeds (22%) making the sample representative of the Danish dairy cow population [15, 16]. For this study each calf was blood sampled once between two and nine days of age. Blood was collected by jugular venipuncture into a 10 mL plain vacuum tube (KRUUSE, Langeskov, Denmark). Blood samples were centrifuged at 4500 rpm for 4 to 10 min and serum pipetted into 5 mL carrier tubes. Serum samples were kept cold for up to 24 h, then frozen at -18 ⁰C and later transported on ice to Aarhus University (Department of Animal and Veterinary Sciences) for analysis. Serum was analysed for total IgG concentration with an ELISA (E11-118, Bethyl Laboratories Inc., Montgomery, TX, USA) according to the manufacturer’s guidelines. %Brix was measured with a digital refractometer (ATAGO Pocket refractometer PAL-1, Tokyo, Japan).

The percentage of calves with FTPI for both serum IgG and %Brix was calculated and the difference between heifers and bulls was compared. Serum IgG concentration and %Brix was compared by Pearson’s correlation coefficient and simple linear regression. All data management and analyses were done in R [17].

A total of 834 calves with 430 bull calves and 404 heifer calves were sampled in the 83 dairy herds. The number of calves sampled distributed by age in days varied from 83 to 122 calves (Table 1), with a mean and median age of 5.4 and 5 days, respectively, and the number of calves sampled from each herd ranged from 1 to 69 (Table 2), with a mean and median of 10.05 and 7 calves, respectively.

**Table 1:** Number of included calves distributed by age and mean/median serum IgG concentration and percentage Brix

	Age (days)							
	2	3	4	5	6	7	8	9
**No. of calves**	95	104	112	118	122	116	84	83
Mean (median) IgG (mg/mL)*	21.00 (19.32)	19.15 (19.17)	18.45 (17.04)	18.91 (18.10)	17.95 (17.11)	16.34 (15.41)	15.33 (13.43)	16.72 (16.12)
Mean (median) %Brix**	8.54 (8.4)	8.51 (8.5)	8.44 (8.5)	8.49 (8.5)	8.44 (8.4)	8.40 (8.4)	8.44 (8.4)	8.37 (8.3)

^*^ Measured by Enzyme-linked immunosorbent assay (ELISA)

^**^ Measured by a digital Brix refractometer

**Table 2:** Number of included calves two to nine days of age from each herd

			No. of calves																			
			1	2	3	4	5	6	7	8	9	10	12	14	17	19	25	44	49	51	59	69
**No. of herds**			6	8	2	4	8	11	5	9	5	12	1	3	1	1	2	1	1	1	1	1

**Fig. 1:**
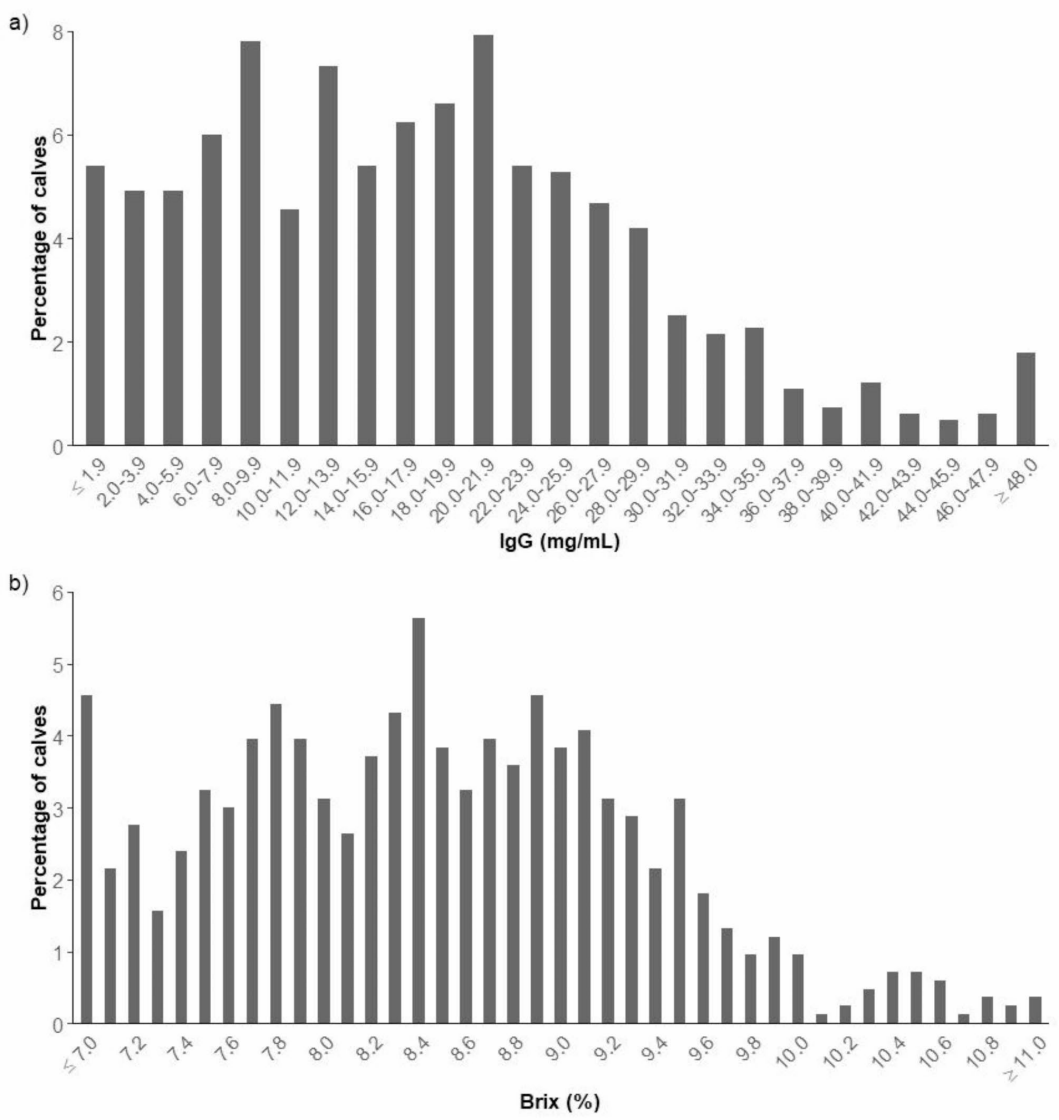
Percentage frequency distributions of (**a**) serum IgG concentration and (**b**) percentage Brix (%Brix). Legend: Serum IgG concentration was measured by Enzyme-linked immunosorbent assay (ELISA) and %Brix by a digital Brix refractometer. A total of 834 Danish dairy calves aged two to nine days were included

Summary statistics for all calves and divided by sex for serum IgG concentration and %Brix, respectively, are shown in Table 3. Serum IgG concentration was not normally distributed with a median value of 17.05. For %Brix the mean value was 8.5%. No statistically significant difference was found between heifer and bull calves for serum IgG concentration and %Brix, respectively. A total of 592 (71.0%) and 242 samples (29.0%) had an IgG concentration ≥ 10 mg/mL and < 10 mg/mL, respectively. For %Brix 541(64.9%) and 293 (35.1%) was ≥ 8.1 and < 8.1%, respectively. Twenty-four samples (2.9%) had an IgG concentration < 1 mg/mL. The percentage frequency distributions of serum IgG concentration and %Brix are shown in Fig. 1.

**Table 3:** Summary statistics for serum IgG concentration and percentage Brix. All calves and divided by sex

		ELISA, IgG (mg/mL)*					Brix refractometer (%)**			
	*n*	Mean	Median	Min - max	Q1 - Q3		Mean	Median	Min - max	Q1 - Q3
**All**	834	18.04	17.05	0.04–60.62	9.03–25.02		8.5	8.4	5.8–13.1	7.8–9.1
**Heifer**	404	18.62^a^	17.80	0.16–60.62	9.40–25.50		8.5^a^	8.4	6.5–10.8	7.8–9.1
**Bull**	430	17.48^a^	16.72	0.04–57.52	8.58–24.77		8.4^a^	8.4	5.8–13.1	7.7–9.0

^*^ Measured by Enzyme-linked immunosorbent assay (ELISA)

^**^ Measured by a digital Brix refractometer

n = Number of calves

T-test: No statistically significant difference (*P* < 0.05) was found between heifer and bull calves indicated by same superscript letters

For serum IgG, percentage of calves in each of the four categories; poor, fair, good and excellent [8] were 29.0%, 23.5%, 22.3% and 25.2%, respectively, and for %Brix 35.1%, 30.9%, 18.5% and 15.5%, respectively.

**Fig. 2:**
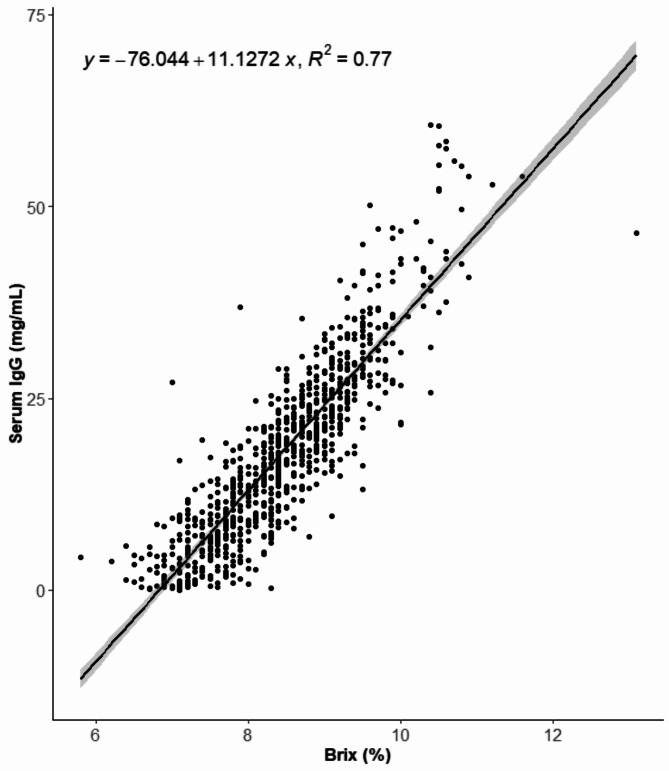
Serum IgG concentration compared with percentage Brix (%Brix) (*r* = 0.88). Legend: Serum IgG concentration and %Brix were measured by Enzyme-linked immunosorbent assay (ELISA) and a digital Brix refractometer, respectively. 834 Danish dairy calves aged two to nine days were included

Serum IgG concentration was found to be highly correlated (*r* = 0.88) with %Brix. The equation from the linear regression model (R^2^ = 0.77) was given by y=-76.0440 + 11.1272x (Fig. 2).

We reported a considerable percentage of calves with FTPI in Denmark (29% and 35.1% for serum IgG and %Brix, respectively). A subsample of the presented data evaluated immunization based on Brix% and disease in 77 heifer calves [18]. FTPI amongst these heifer calves was similar to the present findings at 31% aross the nine included dairy herds. These Danish findings are also in line with Swedish studies, where percentages of calves with FTPI of 31% [19] and FTPI ranging from 20–70% [20] were reported. A novel European study found 39.7% of calves with FTPI based on a cut-off of < 8.4% Brix [21] and a Korean study reported 36% [10]. On the other hand, recent studies conducted in the USA and Canada, reported a lower percentage of calves with FTPI ranging from 4.75-25% [5, 11, 14, 22] and a recent review found a median of 21% (range 1.6-56%) of FTPI among selected studies [23]. There is thus great difference in percentages of calves with FTPI across herds and countries and many factors probably influence the findings.

When applying the recent recommendation on serum IgG levels and %Brix dividing calves into the four categories; poor, fair, good and excellent [8], the percentage of calves in the poor and excellent categories in our study differs considerably from their findings and recommendations. However, for serum IgG their findings are based on RID measures [5]. Though IgG values determined by RID and ELISA are highly correlated [12, 13], the values of RID and ELISA can probably not be directly compared. A study found that serum IgG values based on RID were about 1.8 times higher than those based on ELISA [12] and another study found RID to be on average 8.6 mg/mL higher than ELISA [13]. Similarly, plasma IgG concentrations were significantly lower when measured by ELISA compared to a RID kit although the correlation between the two was weaker in this study (*r* = 0.59) [24]. In addition, cut-off values for FTPI derived from ELISA were found to be 8 mg/mL [25] and 5.4 mg/mL [13]. Contrastingly, a good agreement (94%) was found between the ELISA used in our study and a RID kit (VET-RID) [10]. Although the use of RID kits is disputed, the RID kit VET-RID more accurately estimated the expected IgG values compared to the RID kit SRID [26]. In conclusion, the correlation and difference between corresponding values of the used ELISA and RID are not known, making it difficult to compare the IgG values from the two tests directly. In order to fully understand our findings on serum IgG a direct comparison of ELISA and RID values would be necessary.

The cut-off values for %Brix corresponding to FTPI also varies between studies. One study found the best combination of specificity and sensitivity at ≤ 8.5% Brix [27] which was very close to the < 8.4% Brix found by another study [11]. A lower cut-off has also been reported with the optimal combination at 7.8% Brix equivalent to an IgG concentration of 12 mg/mL [14]. A recent review did, however, find a predominance of studies with a cut-off of < 8.4% Brix [28]. They point out that factors like age and breed, but also type of refractometer and serum storage may influence %Brix measurements and thereby contribute to the differences seen between studies. In addition, the reference method used to determine the cut-off may as well have an impact. We found serum IgG concentration determined by ELISA and %Brix to be highly correlated (*r* = 0.88), which confirms the use of a Brix refractometer as a reliable, on-farm tool. Other studies comparing RID and %Brix found similar levels of correlations; 0.93 [11], 0.87 [14], and 0.79 [27].

The recent study on recommended serum IgG levels excluded all samples with IgG values below 1 mg/mL as unrealistic measurements leading to fewer calves with FTPI (12.0%) [5]. Another study excluded samples below 3.43 mg IgG/mL ending up with 25% of calves with FTPI [14]. Twenty-four of our samples (2.9%) had IgG values below 1 mg/mL, but since our objective was to describe the level of passive immunity in Danish dairy calves, these samples were not considered as outliers.

No statistically significant difference was found serum IgG and %Brix between heifer and bull calves in our study. Based on our results there does not seem to be a general difference in colostrum management between heifer and bull calves in Denmark. Contrastingly, findings from 2008 showed a lower concentration of serum total protein for bull calves compared to heifer calves [29] and colostrum feeding was at a lower volume after the first feeding and took place later for bull calves compared to heifer calves in the US [30].

A study suggested that serum IgG concentration can be indicative of transfer of passive immunity from 24 h after colostrum intake until the calf is nine days old [2]. Under field conditions, where the timing of colostrum allocation may vary and is not known, we found it reasonable to measure serum IgG concentration from day two to nine of age where day zero is the day of birth.

In conclusion, the level of passive immunity in this study of Danish dairy calves appeared below the levels reported elsewhere and recommended by others. However, applying cut-offs based on RID to ELISA-derived serum IgG values are not straightforward and a direct comparison should be interpreted with caution. Cut-off values for %Brix equivalent to FTPI also varies considerably between studies, but we found ELISA and %Brix to be highly correlated. Our study showed no sex difference in FTPI status of calves. More research is warranted to determine ELISA-based FTPI cut-offs and recommendations.

## Data Availability

The datasets used and analysed during the current study are available from the corresponding author on reasonable request.

## Declarations

### Prior publication

Data on %Brix from nine herds including 77 heifer calves were used to assess immunisation status (Otten ND, Skarbye AP, Krogh MA, Michelsen AM, Nielsen LR. Monitoring bovine dairy calf health and related risk factors in the first three months of rearing. Acta Vet Scand. 2023;65:45. 10.1186/s13028-023-00708-8). In addition, data on IgG and %Brix from the same nine herds were published in a master’s thesis where an additional 13 herds were included (Wilm J. Association between serum IgG Level and clinical signs of gastrointestinal disease in newborn Danish dairy calves. Master’s thesis. University of Copenhagen. 2019).
